# Unexpectedly high leprosy seroprevalence detected using a random surveillance strategy in midwestern Brazil: A comparison of ELISA and a rapid diagnostic test

**DOI:** 10.1371/journal.pntd.0005375

**Published:** 2017-02-23

**Authors:** Marco Andrey C. Frade, Natália A. de Paula, Ciro M. Gomes, Sebastian Vernal, Fred Bernardes Filho, Helena B. Lugão, Marilda M. M. de Abreu, Patrícia Botini, Malcolm S. Duthie, John S. Spencer, Rosa Castália F. R. Soares, Norma T. Foss

**Affiliations:** 1 Dermatology Division, Department of Medical Clinics, Ribeirao Preto Medical School, University of São Paulo, Ribeirão Preto, Brazil; 2 Dermatology Division, Department of Medical Clinics, Faculty of Medicine, University of Brasília, Brasília, Brazil; 3 Service of Dermatology, University of Oeste Paulista, Presidente Prudente, Brazil; 4 Infectious Diseases Research Institute, 1616 Eastlake Av. E, Seattle, WA, United States of America; 5 Colorado State University, Department of Microbiology, Immunology and Pathology, Fort Collins, CO, United States of America; 6 General Coordination of Leprosy and Eliminating Diseases, Surveillance Secretariat in Health, Brazilian Health Ministry, Brasília, Distrito Federal, Brazil; Fondation Raoul Follereau, FRANCE

## Abstract

**Background:**

Leprosy diagnosis is mainly based on clinical evaluation, although this approach is difficult, especially for untrained physicians. We conducted a temporary campaign to detect previously unknown leprosy cases in midwestern Brazil and to compare the performance of different serological tests.

**Methods:**

A mobile clinic was stationed at the main bus terminal in Brasília, Brazil. Volunteers were quizzed and given a clinical exam to allow categorization as either patients, known contacts of patients or non-contacts, and blood was collected to determine anti-PGL-I and anti-LID-1 antibody titers by ELISA and by the NDO-LID rapid test. New cases of leprosy and the impact of performing this broad random surveillance strategy were evaluated. Accuracy values and concordance between the test results were evaluated among all groups.

**Results:**

Four hundred thirty-four individuals were evaluated, and 44 (10.1%) were diagnosed with leprosy. Borderline forms were the most frequent presentation. Both tests presented higher positivity in those individuals with multibacillary disease. Serological tests demonstrated specificities arround 70% for anti-PGL-1 and anti-LID ELISA; and arround 40% for NDO-LID. Sensitivities ranged from 48 to 62%. A substantial agreement between NDO-LID and ELISA with concomitant positive results was found within leprosy patients (Kappa index = 0.79 CI95% 0.36–1.22).

**Conclusions:**

The unexpectedly high leprosy prevalence in this population indicates ongoing community-based exposure to *Mycobacterium leprae* antigens and high rates of subclinical infection. All tests showed low specificity and sensitivity values and therefore cannot be considered for use as stand-alone diagnostics. Rather, considering their positivity among MB patients and non-patients, these tests can be considered effective tools for screening and identifying individuals at high risk who might benefit from regular monitoring.

## Author summary

Leprosy is a disease that affects the skin and peripheral nervous system, caused by *Mycobacterium leprae* (*M*. *leprae*), that to survive hides itself within the host cells and grows slowly. Its diagnosis is difficult and requires trained physicians. Laboratorial tests are frequently negative depending on individual response. *M*. *leprae* transmission is mainly man to man, because this is very important diagnosis and treatment of patients for breaking the transmission chain. We conducted a temporary campaign to detect previously unknown leprosy cases in midwestern Brazil and to compare the performance of different serological tests (anti-PGL-I and anti-LID-1 antibody titers by ELISA and by the NDO-LID rapid test). Four hundred thirty-four individuals were evaluated, and 44 (10.14%) were diagnosed with leprosy. Each test presented higher positivity in those individuals with multibacillary disease. For leprosy diagnosis, serological tests demonstrated specificities arround 70% for anti-PGL-1 and anti-LID ELISA; and arround 40% for NDO-LID. Sensitivities ranged from 48 to 62%. Anti-PGL-I and anti-LID-1 ELISA sensitivity values were 30.1% and 23.7% respectively, while NDO-LID sensitivity was 51.6% among contacts and non-contacts. The low specificity and sensitivity values of serological tests establish that they cannot be considered stand-alone diagnostics considering the importance of clinical manifestations in leprosy. Rather, we suggest using such tests as a means to triage potential patients for follow-up clinical examinations.

## Introduction

Leprosy diagnosis is mainly based on clinical evaluation [1]. This approach represents a low-cost strategy; however, it is subjective and dependent upon the experience of the examiner, a difficult task for untrained physicians [2].

Early recognition is critical for the prompt initiation of multidrug therapy (MDT) to prevent irreversible adverse outcomes [1]. Although the number of new cases registered annually by the leprosy surveillance program of the World Health Organization (WHO) have been dramatically reduced since the introduction of multidrug therapy, MDT [3], endemic leprosy pockets linger in countries such as Brazil, India and Indonesia, and current case detection strategies require clinical expertise for diagnosis. Many national leprosy-specific control programs have been integrated into the general health system to facilitate patient care. Nevertheless, these control programs have not reduced the expected number of new cases, as they have been implemented in high endemic countries, where the actual number of cases is 6-8-fold higher than currently reported [4]. The present recommendation is to extend surveillance following the “index” case and to examine close contacts [5], particularly those who are blood relatives of multibacillary (MB) patients [6]. Not only are the logistics of such an approach extremely difficult, such activities are complicated by the need to recognize an index case to trigger them.

Bacilloscopy, histopathological examination, serology and polymerase chain reaction are used to recognize leprosy [7, 8]. Phenolic glycolipid-I (PGL-I) is a specific antigen from *Mycobacterium leprae* (*M*. *leprae*) that induces the formation of IgM class antibodies [9]. Anti-PGL-I antibodies are found in high concentrations in the sera of MB patients [9] and are correlated with the bacterial index [10, 11]. This specific humoral response has been investigated as an auxiliary tool for diagnosis [12, 13], although its utility in active surveillance remains uncertain [14]. Antibody responses to other antigens, such as Leprosy IDRI Diagnostic (LID)-1 protein, a fusion of two well-recognized *M*. *leprae* proteins (ML0405/ML2331) [15], have been studied to improve the accuracy of serological tests [16, 17]. Therefore, the combination of anti-LID-1 IgG and anti-PGL-I IgM detection is a strategy to improve test sensitivity in the entire clinical spectrum of disease [18]. Similarly, NDO-LID, a commercial immunochromatographic lateral flow test, has been developed to detect anti-PGL-I and anti-LID1 antibodies for initial clinical screening [19, 20] and is applied easily in the field with minimal training.

In the current study, we conducted clinical examinations during a brief active campaign in midwestern Brazil to determine the extent of undiagnosed leprosy. In parallel, we tested all individuals using and comparing three leprosy serological methods: anti-PGL-I and anti-LID-1 by ELISA and the NDO-LID commercial rapid diagnostic test (RDT).

## Methods

### Screening

A mobile clinic was established from January 13^th^ to 17^th^, 2014, during a diagnosis campaign in a bus terminal of Brasilia, Federal District (FD), Brazil. After a broad recruitment strategy (radio, television and banner advertisements), individuals were enrolled by health care agents if they presented just any general symptoms of leprosy and/or had knowingly been in contact with someone with leprosy.

### Clinical evaluation

The enrolled individuals underwent a clinical dermatological-neurological exam by dermatologist. Leprosy diagnosis was made by the finding of at least one of the following signs: (A) Definite loss of sensitivity and/or some dysautonomia in a pale (hypopigmented) or reddish skin patch or (B) peripheral nerve impairment with respective loss of sensitivity and/or muscle weakness [1]. All leprosy diagnoses were certified by at least two experts. Leprosy patients were classified based on the Ridley-Jopling classification [21] modified according to the following forms: indeterminate (I), polar tuberculoid (TT), borderline tuberculoid (BT), borderline (BB), borderline lepromatous (BL) and polar lepromatous (LL), and according to WHO operational criteria (paucibacillary, PB (I and TT patients) and multibacillary, MB (Borderline forms and LL patients)), and they were referred to a health unit for standard MDT.

### Laboratory evaluation

Blood was collected and the antibody response was assessed using the commercial RDT (NDO-LID, Orange Life, Rio de Janeiro, Brazil) and also with an antigen-specific antibody ELISA. A specialized immunologist, who was blinded to the patient’s condition, performed the ELISA reactions and analyzed the results.

#### NDO-LID

One drop of total blood (10–20 μl) collected by venopuncture was placed into the well following the manufacturer's instructions. The NDO-LID results were considered positive or negative when the test strip was photographed, read, and analyzed by the Smart Reader application device (NDO-LID SR, Orange Life, Rio de Janeiro, Brazil). Additionally, the NDO-LID results were scored visually by three independent evaluators using qualitative (positive or negative) and semi-quantitative (0, 1+, 2+) scores. The final qualitative results were set when at least two evaluators agreed with a result. The final semi-quantitative results were considered as the mean value from the visual scores of each evaluator.

#### Anti-PGL-I and anti-LID-1 ELISA

ELISA microplate wells were coated overnight with antigens, synthetic PGL-I (12.5 ng/well ND-O-BSA) or LID-1 (50 ng/well) antigen in 0.1M carbonate/bicarbonate pH 9.6 coating buffer (50 μl). Following blocking with blocking buffer (1% bovine serum albumin in phosphate buffered saline pH 7.2 with 0.05% Tween, 1% BSA/PBS/T) for 1 hour, sera were diluted in this same blocking solution, tested at a 1:400 dilution (100 μl) and subsequently incubated for 2-h at room temperature (RT). Then, the wells were washed with PBS with 0.05% Tween20 (PBS/T, wash buffer) six times. Secondary peroxidase-conjugated anti-human IgM (anti-PGL-I) or anti-human IgG (anti-LID-1) (1:20,000, Abcam, Cambridge, UK) was added for another 2-h incubation period. Following this incubation, the wells were washed with PBS/T six times followed by the addition of 100 μl of substrate (3,3′,5,5′-tetramethylbenzidine; TMB). After 15-minutes at RT-incubation, 50 μl of stop solution (H_2_SO_4,_ 1 M) was added. Optical density (OD) values were determined with an ELISA plate reader (Asys Expert Plus-Microplate Reader UK) at 450 nm.

For anti-LID-1 ELISA positivity, the cut-off (0.100) was calculated from the OD average among 18 healthy subjects multiplied by five. For the anti-PGL-I ELISA, the cut-off (0.608) was based on the OD average among healthy subjects multiplied by 2.1 plus 10%, as previously described [15, 22].

In both ELISAs, the respective index was calculated by dividing the OD of each sample by the cut-off [15, 22], and indexes above 1.0 were considered positive.

### Statistical analysis

Categorical variables between the groups were compared using the Chi-square (χ^2^) test. Fisher’s exact test was used when data were sparse. Using the NDO-LID result as a reference, an inter-rater agreement between the tests was performed (Kappa index: <0 = no agreement, 0–0.19 = poor, 0.2–0.39 = fair, 0.4–0.59 = moderate, 0.6–0.79 = substantial, and 0.8–1.0 = almost perfect agreement); this approach was also applied to compare the NDO-LID results by the Smart Reader and by visual reading. Significance was set at *p*<0.05, with a 95% confidence interval, using two-tailed comparisons. The statistical analysis was performed using OpenEpi version 3.01 (Emory University, Rollins School of Public Health, Atlanta, Georgia, USA). Missing variables were ignored, and only patients who underwent all exams were tested for agreement.

### Ethics

Patients were included after signing the informed consent form. Parents/guardians provided informed consent on behalf of all child participants. The study complies with the Declaration of Helsinki, as revised in 2013. This study was approved by the Ethics Committee of the Clinical Hospital of Ribeirão Preto (HCRP N° 16620/2014).

## Results

**Fig 1:**
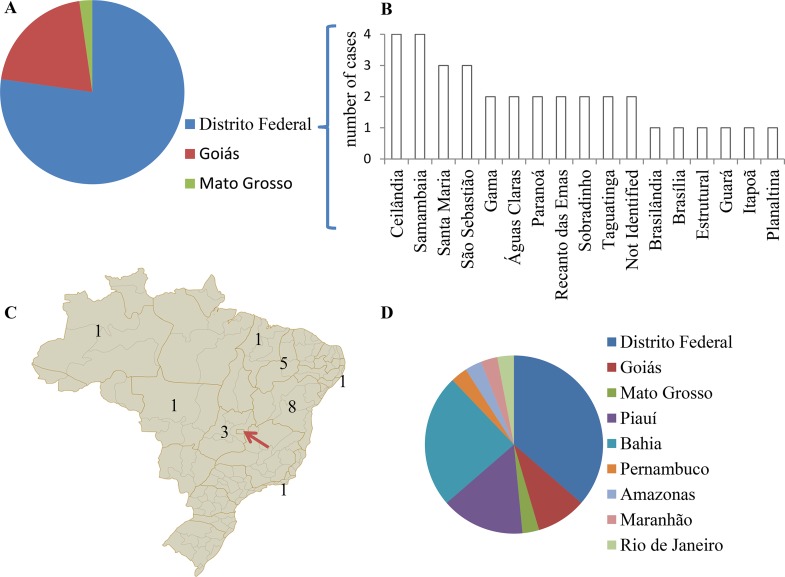
Residency history of leprosy cases identified in the temporary clinic. The 44 leprosy cases identified during the surveillance period, reported their residency based on state. Red arrow: Federal district. (A)—At the time of survey, 34 were resident in the Federal District, 9 in neighboring Goiás and 1 was living in Mato Grosso. (B)—For patients residing in the Federal District, further delineation was made to their current administrative region. (C and D)—Each case identified their state of origin.

A total of 434 individuals were evaluated, and 44 (10.1%) were diagnosed with leprosy. According to the WHO operational classification, 88.6% were classified as MB. The Borderline forms were the most represented, with 36 cases (81.8%; Fig 1; Table 1) distributed among BT (n = 5), BB (n = 24) and BL (n = 7) patients. Blood collection was performed on 381 individuals, while 53 individuals declined, including a confirmed leprosy patient. Table 2 shows the individual demographics.

**Table 1:** Clinical characterization of leprosy patients and healthy individuals (contacts or non-contacts).

Clinical Form (Leprosy patients)	N	Positive N (%)		
		a-PGL-I	a-LID-1	NDO-LID SR
Indeterminate (I)	3	2 (66.7)	1 (33.3)	0 (0.0)
Tuberculoid (TT)	2	0 (0.0)	2 (100)	2 (100)
Borderline Tuberculoid (BT)	5	3 (60.0)	3 (60.0)	4 (80.0)
Borderline Borderline (BB)	23	11 (47.8)	7 (30.4)	12 (52.2)
Borderline Lepromatous (BL)	7	5 (71.4)	5 (71.4)	6 (85.7)
Lepromatous (LL)	3	3 (100)	3 (100)	3 (100)
TOTAL	**43**	**24 (55.8)**	**21 (48.8)**	**27 (62.8)**
MB	38	22 (57.9)	18 (47.4)	25 (65.8)
PB	5	2 (40)	3 (60)	2 (40)
TOTAL	**43**	**24 (55.8)**	**21 (48.8)**	**27 (62.8)**
Contact History (Healthy individuals)				
Contacts	93	28 (30.1)	22 (23.7)	48 (51.6)
Non-contacts	245	84 (34.3)	67 (27.3)	148 (60.4)
TOTAL	**338**	**112 (33.1)**	**89 (26.3)**	**196 (58.0)**

a-PGL-I = Anti-PGL-I ELISA. a-LID-1 = anti-LID-1 ELISA. NDO-LID SR = NDO-LID results from the Smart Reader application device

**Table 2:** Characterization of the individuals and patients included in the study.

	Healthy Individuals (N = 390) N (%)	Patients with clinical diagnosis of Leprosy (N = 44) N (%)	P-value
Sex			0.109
Male	165 (42.3)	13 (29.5)	
Female	225 (57.7)	31 (70.5)	
Age mean (range)	46 (1–87)	42.1(9–73)	0.232
Under 15 years old	25 (6.4)	2 (4.5)	
15 years old and older	365 (93.6)	42 (95.5)	
State of residency			0.110
Federal District	338 (86.7)	34 (77.3)	
Other State	52 (13.3)	10 (22.7)	

Among the leprosy patients, 34 resided within FD (77.3%), whereas the remaining patients lived in either neighboring Goiás (n = 9) or Mato Grosso State (n = 1) (Fig 1A). Cases in the FD were distributed throughout at least 16 different administrative regions, with the most cases from any one administrative region being only 4 (Fig 1B). Furthermore, although more than half were originally from other states and had migrated into the Federal District (Fig 1C), the majority of patients had been in residency for extended periods of time. The 44 patients had a mean time of residency in their administrative regions of 13.5 years, and 32 of them had lived more than 5 years in the same place.

Twenty-two (50%) of the leprosy patients did not report any known leprosy patient contact. Among the individuals without any clinical signs of disease (n = 390), 99 (25.4%) had a leprosy contact, whereas 291 (74.6%) declared no known contact.

Regarding the blood samples collected from healthy individuals (n = 338), 93 (27.5%) were contacts of persons with leprosy, whereas 245 (72.5%) did not have any known contact. The results of the anti-PGL-I and anti-LID-1 ELISA and the NDO-LID are characterized for each clinical form in Table 1. In leprosy diagnosis, considering all disease forms, each of the tests demonstrated a specificity of approximately 70% for anti-PGL-1 and anti-LID ELISA and of approximately 40% for NDO-LID. Sensitivity was less than 65% in all tested techniques. (Table 3). Considering only healthy subjects (n = 338), with the aim of identifying leprosy contacts (Table 3), the sensitivy values of anti-PGL-I and anti-LID-1 ELISA were 30.11% and 23.66% respectively, while NDO-LID sensitivity was 51.61%. Specificity values were lower than 75% in all the three tests.

**Table 3:** Accuracy of diagnosis of serological tests for leprosy.

	a-PGL-I % (CI 95%)	a-LID-1% (CI 95%)	NDO-LID SR % (CI 95%)
	**Leprosy Patients and Healthy Individuals**		
Sensitivity	55.81% (41.11–69.57)	48.84% (34.62–63.25)	62.79% (47.86–75.62)
Specificity	66.86% (61.68–71.67)	73.67% (68.73–78.08)	42.01% (36.87–47.33)
Positive Predictive Value	17.65% (12.16–24.92)	19.09% (12.84–27.43)	12.11% (8.46–17.04)
Negative Predictive Value	92.24% (88.21–94.98)	91.88% (88.01–94.58)	89.87% (84.18–93.67)
	**Contacts and Non-contacts**		
Sensitivity	30.11% (21.73–40.07)	23.66% (16.17–33.23)	51.61% (41.60–61.50)
Specificity	65.71% (59.57–71.37)	72.65% (66.75–77.85)	39.59% (33.67–45.83)
Positive Predictive Value	25.00% (17.90–33.76)	24.72% (16.93–34.60)	24.49% (19.00–30.96)
Negative Predictive Value	71.24% (65.02–76.75)	71.49% (65.58–76.73)	68.31% (60.26–75.39)

a-PGL-I = Anti-PGL-I ELISA. a-LID-1 = anti-LID-1 ELISA. NDO-LID SR = NDO-LID results from the Smart Reader application device

We observed a substantial agreement between the NDO-LID and antibody-detection ELISA (anti-PGL-I plus anti-LID-1), with a κ index value of 0.79 (CI 95% 0.36–1.22) for the leprosy patients. A fair agreement (κ index between 0.19 and 0.20) was shown between the NDO-LID and ELISA assays, with concomitant results between individuals with known contact and those without. NDO-LID and the anti-PGL-I ELISA showed a moderate agreement (0.37 κ index value (CI 95% 0.08–0.67)) and a fair agreement when the NDO-LID was compared with the anti-LID-1 ELISA (0.35 CI95% (0.06–0.63)) among the leprosy patients.

We would expect the NDO-LID titers to be represented by the reactivity to each individual component, i.e., approximating the combination of the anti-PGL-I and the anti-LID-1 titers by ELISA. The ELISAs were stratified by indices (Tables 4 and 5), and the results were compared between the results obtained by the Smart Reader and by visual reading. For ELISA indices <1.0, there was much lower concordance with the response given by Smart Reader; however, for ELISA indices >1.0, there was better agreement with the Smart Reader results. Further, there was moderate agreement (0.55 κ index (CI 95% 0.45–0.66)) between the results read with Smart Reader and the results by visual interpretation (Tables 4 and 5).

**Table 4:** Distribution the positivity of NDO-LID test according the distribution of the ELISA assay index (I).

	Healthy Individuals							Patients			
**Index Range (I) a-PGL-I (N)**	**NDO-LID SR**	**% Concordance**	**NDO-LID Visual**			**% Concordance**	**Kappa Index (SR x Visual)**	**I**	**T**	**B**	**L**
	**(Positive)**		**(Positive)**								
			**+**	**++**	**Total**						
**0.0 ≤ I <1.0** (226)	102	45.1	71	9	80	35.5		1	2	15	0
**1.0 ≤ I <1.5** (65)	51	78.5	31	17	48	73.8		1	0	8	0
**1.5 ≤ I <2.0** (27)	24	88.9	10	10	20	74.1		1	0	6	0
**I ≥2.0** (20)	19	95	7	10	17	85		0	0	6	3
**Index Range (I) a-LID-1 (N)**	**NDO-LID SR**	**% Concordance**	**NDO-LID Visual**			**% Concordance**	0.55 (0.44–0.66)	**I**	**T**	**B**	**L**
	**(Positive)**		**(Positive)**								
			**+**	**++**	**Total**						
**0.0 ≤ I <1.0** (248)	139	56	89	27	116	46.8		2	0	20	0
**1.0 ≤ I <1.5** (48)	33	68.8	14	13	27	56.3		1	2	6	0
**1.5 ≤ I <2.0** (17)	10	58.8	7	3	10	58.8		0	0	4	0
**I ≥2.0** (25)	14	56.0	7	4	11	44		**0**	**0**	**5**	**3**

a-PGL-I = Anti-PGL-I ELISA. a-LID-1 = anti-LID-1 ELISA. NDO-LID SR = NDO-LID results from the Smart Reader application device SR = Smart Reader application device; I = Indeterminate, T = Tuberculoid, B = Borderline, L = Lepromatous

**Table 5:** Inter-rate agreement between tests in leprosy patients, known contacts and non-contacts.

	a-PGL-I (+) N (%)	a-PGL-I (-) N (%)	Kappa Index (CI 95%)	a-LID-1 (+) N (%)	a-LID-1 (–) N (%)	Kappa Index (CI 95%)	a-PGL-I (+) and a-LID-1 (+) N (%)	a-PGL-I (+) and a-LID-1 (-) N (%)	a-PGL-I (-) and a-LID-1 (+) N (%)	a-PGL-I (-) and a-LID-1 (-) N (%)	Kappa Index (CI 95%)
	**Leprosy Patients**										
**NDO-LID SR (+)**	19 (44.2)	8 (18.6)		17 (39.5)	10 (23.3)		11 (25.5)	8 (18.6)	6 (14.0)	2 (4.7)	
**NDO-LID SR (-)**	5 (11.6)	11 (25.6)	0.37 (0.08–0.67)	4 (9.3)	12 (27.9)	0.35 (0.06–0.63)	0 (0)	5 (11.6)	4 (9.3)	7 (16.3)	0.79^a^ (0.36–1.22)
**Total**	**43**			**43**			**43**				
	**Known contacts**										
**NDO-LID SR (+)**	22 (23.7)	26 (28.0)		13 (13.9)	35 (37.6)		6 (6.5)	16 (17.2)	7 (7.5)	19 (20.4)	
**NDO-LID SR (-)**	6 (6.5)	39 (41.9)	0.32 (0.13–0.50)	9 (9.7)	36 (38.7)	0.06 (-0.10–0.23)	2 (2.2)	4 (4.3)	7 (7.5)	32 (34.4)	0.19^a^ (0.01–0.39)
**Total**	**93**			**93**			**93**				
	**Non-contacts**										
**NDO-LID SR (+)**	72 (29.4)	76 (31.0)		44 (18.0)	104 (42.5)		23 (9.4)	49 (20.0)	21 (8.6)	55 (22.4)	
**NDO-LID SR (-)**	12 (4.9)	85 (34.7)	0.32 (0.21–0.43)	23 (9.4)	74 (30.2)	0.04 (-0.05–0.15)	6 (2.5)	6 (2.5)	17 (6.9)	68 (27.8)	0.21^a^ (0.08–0.33)
**Total**	**245**			**245**			**245**				

^a^Kappa Index Calculated between a-PGL-I positive Plus a-LID-1 positive and a-PGL-I negative Plus a-LID-1 negative. a-PGL-I = Anti-PGL-I ELISA. a-LID-1 = anti-LID-1 ELISA. NDO-LID SR = NDO-LID results from the Smart Reader application device

## Discussion

According to the leprosy prevalence rate, FD has been considered a non-endemic area since 2005 [23]; however, the present recruitment procedure resulted in a different scenario with a very high percentage of new cases of leprosy, similar to what one would expect from a hyperendemic area [12, 24]. This fact can be explained by an expected high sensitivity in the screening strategies incorporating our passive random surveillance strategy. Importantly, the leprosy cases detected were not recent emigrants from areas recognized as being hyper-endemic for leprosy and thus, both our clinical data and serological evaluations indicate that leprosy remains an issue, and that *M*. *leprae* transmission continues, in FD.

Leprosy diagnosis is still essentially defined by clinical examination. However, in many cases, the changes can be subtle and are often missed even by specialists. Bacilloscopy, histopathology and serology tests can be used to assist the clinical diagnosis and are also useful in spectral and treatment categorization [25, 26]. Nevertheless, the accuracy of these tests is still debated, and many groups are searching for simple ways to make them more reliable, especially in the identification of leprosy cases among household contacts [15].

NDO-LID was shown to be positive in 62.8% of the clinically diagnosed leprosy patients, although this rapid test presented a lower specificity than anti-PGL-I and anti-LID-1 ELISA, as described in Table 3. Despite being able to identify the dominant responses to both glycolipid (IgM anti-PGL-I) and protein (IgG anti-LID-1), NDO-LID has the same limitation as the previously used RDT, showing a difficulty in screening patients in early stages of disease and/or PB forms. Regardless, the use of these tests in conjunction with, or as a trigger for, clinical examination may promote earlier detection and treatment of leprosy cases.

Our anti-PGL-I ELISA findings were similar to previously described results [13, 27, 28]. The anti-PGL-I ELISA was able to identify all LL cases (n = 3), 54.3% of borderline forms (n = 19), especially BL patients (71.4%) and 66.7% of I form. Individuals with the TT form (n = 2) were not identified. The anti-LID-1 ELISA results showed a lower percentage of positivity in borderline forms (n = 15/35, 42.9%) and indeterminate forms (n = 1/3, 33.3%). Also in borderline patients, this test performed better in BL forms, detecting a similar percentage of patients (71.4%) in comparison to anti-PGL-I ELISA. However, considering the limited number of samples tested, our data showed 100% positivity in both LL and TT forms (n = 3 and 2, respectively). In general, the three tests performed better in the identification of MB patients, especially in the BL and in the LL forms. Many studies showed that borderline and LL patients produce high titers of IgM against PGL-I, whereas Tuberculoid patients have low levels of specific antibody [9]. Serological tests are expected to show better responses in MB; however, such improvements remain challenging.

Vaz Cardoso et al. [20] found an anti-PGL-I sensitivity from 69.8% to 92.3% in borderline patients in contrast to our results (54.3%). These differences may be explained by recruiting differences. Although we expected a higher sensitivity in a screening campaign, we used a cross-sectional/cohort strategy in which the diagnosis of controls was previously unknown. This approach generated a more pragmatic environment, reducing sensitivity values.

In addition to the importance of achieving an early diagnosis, a systematic and qualified approach to monitoring household contacts is considered essential for breaking the transmission of *M*. *leprae* [5, 29]. Individuals in close contact with untreated MB patients have a 10% lifetime risk of eventually succumbing to leprosy [2, 9, 29–31], particularly when they are consanguineous relatives [29]. Although a positive titre to anti-PGL-I is an infection biomarker, it is not an absolute indicator of disease. Evidence indicates that substantial increases in titers to anti-PGL-I and anti-LID-1 are important indicators of progression, even in the absence of skin lesions or nerve damage [11, 15, 32]. In healthy subjects, a high anti-PGL-I and anti-LID-1 titre suggests that there is a hidden bacillary load driving the response to this antigen. Household contacts demonstrating substantial increases in titre against LID-1 and/or PGL-I should be closely monitored [29, 31].

Worobec et al. [30] reported subclinical *M*. *leprae* infection in endemic regions with anti-PGL-I antibody detected in 1.7 to 35% of all individuals. Thus, the high percentage of positivity found in our group may reflect a situation of occult prevalence with many foci of infection in the municipality because many patients may be unknown contacts of leprosy patients. Therefore, considering this rate of exposure, it was not surprising that the rates of positivity in the contact group for all three tests (52% for NDO-LID, 30% for anti-PGL-I and 24% for anti-LID-1) were fairly similar to those among individuals with no history of contact with a leprosy patient, indicative of the generally high rate of exposure in this region.

Among healthy individuals, the concordance of NDO-LID with both the Smart Reader and the visual analysis with the anti-PGL-I ELISA results increased as indices rose (until 95%). Among healthy individuals, the strongest positive responses with NDO-LID showed the best concordance with individuals with the highest anti-PGL-I ELISA index, reaching a maximum where the calculated index was >2.0 (κ = 95%). In general, individuals with very strong positive responses by NDO-LID or with the highest indices in the ELISA, particularly against LID-1, should receive more careful scrutiny during their clinical examination, and regular follow-up examinations would appear prudent.

The tests evaluated in this study demonstrated low specificity and sensitivity values, which would limit their use as stand-alone tests for leprosy diagnosis. However, the tests do appear useful for screening populations as a way to assist clinicians in patient categorization, contacts’ follow-up of patients with high titers, and to assess the risk of treatment failure. Further examination of the use of serological tests for the early diagnosis of leprosy is warranted to attempts break the cycle of *M*. *leprae* transmission. This is particularly meaningful given our clinical evidence that *M*. *leprae* infection and leprosy remain as a public health concern in FD, a region officially reported having controlled leprosy.

## Conclusion

The unexpected high percentage of leprosy cases and the high number of individuals with positive serological responses to *M*. *leprae* antigens found in this population, previously considered ‘nonendemic,’ revealed a greater than expected extent of subclinical and frank disease, as well as a continuous contact with *M*. *leprae* antigens. These data may point to faults in the health care delivery system and a lack of training by physicians working in the basic health units. Ultimately, more aggressive targeted surveillance strategies, particularly focusing on cities that may experience a large migration of people, should be considered part of a national coordinated leprosy control program.

The positivity of approximately 60% of the anti-PGL-I ELISA and NDO-LID demonstrated that each test is a potential tool to identify suspected patients. The unexpectedly high rates of positivity with NDO-LID among household contacts (50%) and non-contacts (60%) indicates the difficulties in using a single test for diagnosing leprosy.

Despite the limited sensitivity found in all of the diagnosed cases, the anti-PGL-I ELISA and NDO-LID were in complete agreement in identifying all LL and more than 50% of the borderline forms, demonstrating a possible positive impact and the relevance of this type of surveillance campaign to ultimately achieving a reduction in transmission of this disease.

Further studies are necessary to elucidate the efficacy of these serological tests in other diagnostic reference standards; nevertheless, the training of local health professionals to recognize signs of disease and epidemiological surveillance remains the primary strategy for leprosy control.

## Supporting information

S1 Checklist STROBE Statement. Checklist of items that should be included in reports of cross-sectional studie.
(DOC)

S1 Data Sheet Excel with campaign’s data used for analysis. (XLSX)
